# Venetoclax-associated bullous dermatitis: A BCL-2 inhibitor-linked autoimmune blistering disease

**DOI:** 10.1016/j.jdcr.2026.06.035

**Published:** 2026-06-20

**Authors:** Thibaut Delahaye, Patrick Collins, Joan Somja, Arjen F. Nikkels

**Affiliations:** a Department of Dermatology, CHU du Sart Tilman, University of Liege, Liege, Belgium; b Department of Dermatopathology, CHU du Sart Tilman, University of Liege, Liege, Belgium

**Keywords:** acral bullae, BCL-2 inhibitor, bullous dermatitis, chronic lymphocytic leukemia, drug-induced bullous dermatitis, immune dysregulation, venetoclax

## Introduction

Bullous dermatitis (BD) represents the most frequent autoimmune blistering skin disorder, and medications are implicated in up to one-third of cases.^1^ The clinical presentation often resembles that of bullous pemphigoid (BP). Several B-cell–directed oncologic therapies, including Bruton tyrosine kinase inhibitors, anti-CD20 antibodies, and immune checkpoint inhibitors, have been associated with BD through mechanisms of immune dysregulation.

Venetoclax is an oral selective B-cell lymphoma 2 inhibitor widely used as monotherapy or in combination for chronic lymphocytic leukemia (CLL) and small lymphocytic lymphoma.^2^

We report a patient with CLL on long-term ibrutinib who developed multiple acral sanguineous BD lesions shortly after the introduction of venetoclax.

## Case report

A 68-year-old woman with CLL, Binet A since 2005, had been treated with oral ibrutinib 280 mg/day for 7 months without any cutaneous adverse effects. Because the CLL did not respond adequately to ibrutinib, oral venetoclax (400 mg/day) was added using a standard weekly dose-escalation schedule.

Four weeks later, the patient developed painful, tense, large sanguineous bullae strictly confined to the hands (Fig 1, *A*) and feet (Fig 1, *B*), without mucosal involvement. She reported no new medications, infections, or vaccinations. Blood tests did not reveal eosinophilia.

**Fig 1:**
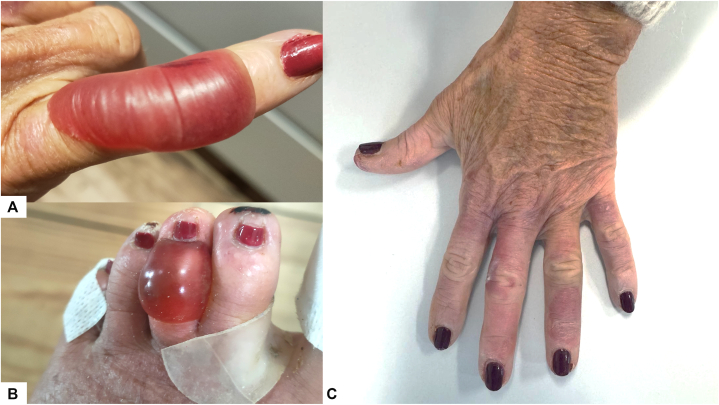
**A,** Tense sanguinous BD lesions of the finger, **(B)** of the toe, **(C)** clinical aspect after 3 dupilumab injections. *BD*, Bullous dermatitis.

Dermatologic examination showed multiple tense sanguineous bullae and postbullous erosions on a mildly erythematous acral background. There was an initial clinical suspicion of BP. A punch biopsy from the edge of a fresh blister demonstrated subepidermal splitting with eosinophils and neutrophils, compatible with BP (Fig 2, *A* and *B*). However, direct immunofluorescence did not show linear immunoglobulin (Ig) G, IgA, IgM, or C3 deposition along the basement membrane zone rather favoring a diagnosis of BD. BP180/BP230 enzyme-linked immunosorbent assay testing was negative and no eosinophilia was noted. The clinic-pathological correlation finally led to the diagnosis of a drug-induced bullous dermatitis (DIBD).

**Fig 2:**
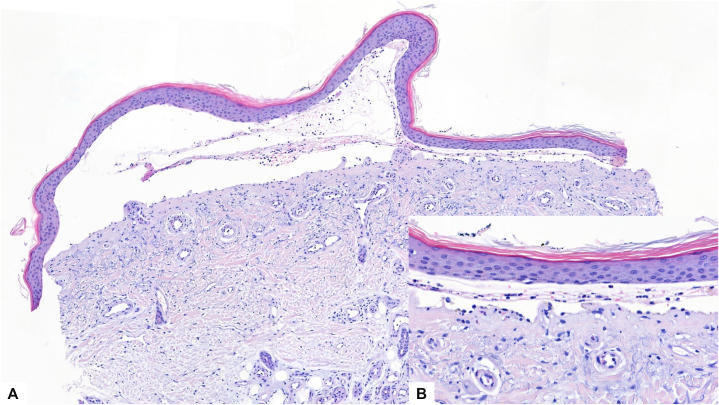
**A,** BD histology, (H/E, 50×), **(B)** high magnification (H/E, 400×). *BD*, Bullous dermatitis.

Given her 7 uneventful months on ibrutinib and the close temporal association with venetoclax initiation, venetoclax-associated BD was considered the most likely origin of the eruption. However, venetoclax was maintained for hematologic reasons.

According to National Comprehensive Cancer Network guidelines for the management of immunotherapy-related toxicities, high-potency topical corticosteroids (clobetasol propionate 0.05% cream once daily) were initiated, along with subcutaneous dupilumab (initial dose 300 mg × 2, followed by 300 mg every 2 weeks) as a steroid-sparing option without drug-interaction concerns. The cutaneous lesions progressively resolved in about 3 to 4 weeks.

At a follow-up 3 months after diagnosis, only postinflammatory hyperpigmentation was noted, and no new cutaneous lesions had appeared (Fig 1, *C*). Tolerance of dupilumab was excellent and venetoclax was continued without further cutaneous events.

## Discussion

Drug-induced bullous pemphigoid and DIBD are increasingly encountered and recognized as a specific group of adverse events. Well-known medication triggers include diuretics, neurologic agents, antibiotics, and immune checkpoint inhibitors.^1^ More recently, several B-cell–directed therapies have been implicated. Bruton tyrosine kinase inhibitors such as ibrutinib and acalabrutinib have been associated with BP since 2018,^3^^,^^4^ and paradoxical cases have been reported with rituximab. These observations suggest that disturbances in B-cell homeostasis may interfere with tolerance to BP180 and BP230 antigens in the basement membrane.

To date, no prior reports have associated venetoclax with BD. This case expands the spectrum of B-cell–active agents capable of inducing autoimmune blistering disease.

Several arguments support venetoclax as the most likely trigger. First, ibrutinib-related BD typically appears early, whereas our patient remained asymptomatic for 7 months on ibrutinib. Second, blistering developed 4 weeks after initiating venetoclax, a typical latency for DIBD. Third, no other triggers—such as new medications, vaccines, or infections—were identified. Finally, venetoclax profoundly remodels B-cell compartments and may disrupt immunologic tolerance.^5^^,^^6^ Together, these factors strongly favor venetoclax rather than ibrutinib as the culprit.

Although venetoclax is not classically associated with autoimmunity, several mechanisms may explain the presentation. Venetoclax induces rapid apoptosis of both malignant and normal B cells, including interleukin-10–producing regulatory B cells. Loss of B-regulatory function may impair interleukin-10–mediated tolerance, enhance Th2 polarization, increase IgG4 class switching, and promote autoreactive clones—mechanisms overlapping with those implicated in BP. In addition, venetoclax promotes mitochondrial apoptosis with features of immunogenic cell death,^7^ leading to the release of damage-associated molecular patterns such as high-mobility group box 1 and adenosine triphosphate. These signals enhance antigen presentation and may unmask basement membrane antigens. Furthermore, abrupt B-cell depletion followed by repopulation may transiently increase B-cell activating factor levels and modify T-cell co-stimulation. Similar mechanisms have been proposed in rituximab-related paradoxical autoimmunity.

Bruton tyrosine kinase inhibitors may induce BD through Th2-skewed immune responses and altered B-cell/T-cell cross-talk. Anti-CD20 therapies can trigger paradoxical autoimmunity via B-reg depletion, providing a mechanistic parallel to venetoclax. Immune checkpoint inhibitors, although acting through different pathways, similarly disrupt immune tolerance and may unmask BP. Venetoclax conceptually fits within this group of therapies capable of resetting B-cell and T-cell regulatory networks.

Acral-predominant BD has been increasingly described in drug-associated cases. The strictly acral distribution in our patient is consistent with a DIBD phenotype and supports a medication-related trigger rather than classic idiopathic BP. Because venetoclax could not be discontinued for hematologic reasons, treatment focused on local control. High-potency topical corticosteroids successfully stabilized the eruption, and dupilumab, shown to be beneficial in BD, including drug-induced forms^8^, ^9^, ^10^ was initiated as a steroid-sparing option.

Another hypothesis is that the addition of venetoclax to ongoing ibrutinib therapy altered immunologic tolerance to ibrutinib, enabling the development of BD.

In conclusion, this case of BD temporally associated with venetoclax illustrates a plausible drug-related mechanism supported by chronology, absence of alternative triggers, and biologic plausibility. Given the widespread use of venetoclax, clinicians should be aware of this potential association and promptly evaluate new acral blistering eruptions to ensure appropriate management while maintaining essential oncologic therapy. Dupilumab seems to be a well-tolerated and efficacious agent for mitigating DIBD without interfering with oncologic therapies.

### Declaration of generative AI and AI-assisted technologies in the writing process

During the preparation of this work the authors used AI-assisted tools in order to refine American English grammar. After using this tool/service, the authors reviewed and edited the content as needed and take full responsibility for the content of the publication.

## Conflicts of interest

None disclosed.
